# The Kinetics of Microcirculatory Dysfunction During Paclitaxel Application in an In Vivo Mouse Model

**DOI:** 10.3390/jcm14144815

**Published:** 2025-07-08

**Authors:** Susanne Reuter, Rika Bajorat, Fabian Müller-Graf, Amelie R. Zitzmann, Stephan H. Böhm, Daniel A. Reuter, Brigitte Vollmar

**Affiliations:** 1 Institute for Experimental Surgery, University Medical Center Rostock, Schillingallee 69a, 18057 Rostock, Germany; brigitte.vollmar@med.uni-rostock.de; 2 Department of Gynecology, University Medical Center Hamburg-Eppendorf, Martinistraße 52, 20246 Hamburg, Germany; 3 Department of Anesthesiology, Intensive Care Medicine and Pain Therapy, University Medical Center Rostock, Schillingallee 35, 18057 Rostock, Germany; rika.bajorat@med.uni-rostock.de (R.B.); fabian.mueller-graf@med.uni-rostock.de (F.M.-G.); amelie.zitzmann@med.uni-rostock.de (A.R.Z.); stephan.boehm@med.uni-rostock.de (S.H.B.); daniel.reuter@med.uni-rostock.de (D.A.R.)

**Keywords:** chemotherapy-induced peripheral neuropathy (CIPN), intravital fluorescence microscopy, kinetics of microvascular damage, endothelial lesions, inflammation

## Abstract

**Objective**: Chemotherapy-induced peripheral neuropathy often has a lasting impact on the quality of life without existing causal treatment options. The aim of this study was to systematically investigate the temporal occurrence of paclitaxel-induced peripheral microcirculatory dysfunction. **Methods**: Thirty-one female SKH-1 mice received six cycles of paclitaxel intraperitoneally in the treatment group and six cycles of saline in the control group. Intravital fluorescence analyses were performed in the groups 180 min after saline administration and immediately, 60 min, 120 min, and 180 min after paclitaxel administration to evaluate the effects on microcirculation and inflammation. **Results**: In addition to signs of systemic inflammation, the intravital microscopy revealed a marked reduction in functional capillary density, increased venous leukocyte adhesion, and endothelial permeability that persisted for at least three hours in paclitaxel-treated mice. **Conclusions**: Our results show that paclitaxel-induced microcirculatory disturbances manifest immediately after application and last at least for 3 h. This suggests that options for prevention or at least amelioration could potentially be most effective if initiated parallel to the induction of chemotherapy and continued for a prolonged period of at least 3 h. Whether and to what extent the prolongation of the preventive strategies influences CIPN in the long term needs to be studied further.

## 1. Introduction

Besides curative oncological therapy, one of the most important treatment goals for cancer patients is the maintenance of the best possible quality of life [1]. A frequent and very serious side effect of chemotherapy with lasting impact on patients’ everyday lives is CIPN. CIPN is described as the most common neurotoxic side effect of chemotherapeutic agents with nerve-damaging potency. There are several drugs that are associated with the development of both acute and chronic CIPN [2,3]. Substances such as platinum derivatives, vinca alkaloids, taxanes, proteasome inhibitors, and other immune inhibitors exhibit a variety of different neurological side effects, which depend on the cumulative dose, the frequency of application, and the individual therapeutic agents [4,5,6,7]. Occasionally these side effects are so severe that they become the dose-limiting factor for primary tumor therapy [2,8]. For example, for paclitaxel, a widely used chemotherapeutic agent, a CIPN prevalence of up to 87% has been described [9]. Up to 30% of these patients develop long-term symptoms that severely impair their quality of life [3,9]. The neurological symptoms can manifest in a variety of ways ranging from sensory symptoms to motor deficits [6]. They can be accompanied by autonomic symptoms, such as paresthesia, allodynia, cold dysesthesia, predominantly distal paresis, and, rarely, by hemodynamic dysregulation [3]. Due to the preferential damage of the long axons, a so-called glove–sock CIPN distribution pattern occurs [10,11]. Currently, there is neither a preventive nor a causative therapy for CIPN [6]. In addition to these neurological symptoms, up to 50% of patients treated with paclitaxel suffer from an acute, transient, primarily muscular and skeletal pain syndrome after the administration of the drug [12]. This syndrome is not clearly attributable to nerve damage, but it shares many clinical features with CIPN. Newer drugs such as duloxetine alone or in combination with pregabalin help reduce the painful sensations of myalgia and arthralgia; however, they do not seem to improve the neurological symptoms of CIPN itself [13]. Patients suffering from this pain syndrome have the highest prevalence of developing CIPN [14,15].

For any preventive or therapeutic approach to CIPN, it is of fundamental importance to know in which timeframe of chemotherapy such an approach should be initiated. This requires the knowledge of when the chemotherapeutic agent itself or the pathophysiological reaction caused by the agent begins to become active at the target structure and when this is resolved. Recently, our group described that paclitaxel—beside its direct neurotoxic effects—causes not only severe microcirculatory disturbances but also profound inflammatory reactions in the terminal vascular bed, most probably co-responsible for the development and severity of CIPN. Thus, the aim of this current investigation was to describe the onset and progress of microcirculatory and inflammatory changes after the application of paclitaxel as surrogates for its timeframe of pharmacological side-effect action [16,17,18,19,20].

## 2. Materials and Methods

This investigation was part of a larger experimental project registered with and approved by the governmental authority (State Office for Agriculture, Farming, Food Security, and Fishing, Mecklenburg-Vorpommern, Germany, file number 7221.3-1-016/20). Independent parts of this project have been published recently [20].

### 2.1. Animal Model

All the animal experiments were executed in accordance with the protection of animals act of Germany [21] and the European Directive 2010/63/EU [22]. As described in detail recently, we used, in total, 31 8-week-old homozygous female SKH1-hr hairless mice weighing approximately 25 g [20]. All the animals were housed in groups of four to five with a 12 h light–dark cycle under standardized conditions of 21 ± 3 °C and about 60% relative humidity, with steady access to water and food ad libitum. The animals received bedding and building materials to set up their environment. The hairless mouse ear model was used for the intravital fluorescence microscopy (IVM) study of skin microcirculation. Due to a genetic defect on chromosome 14, the animal loses its fur after postnatal day 10, making the pinna an easily accessible site of peripheral microcirculation for intravital microscopy of the vessels [23,24].

**Figure 1:**
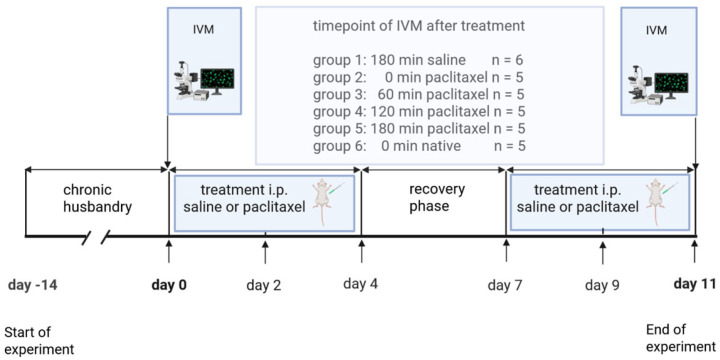
Schematic drawing of the experimental design. The cohort of 31 animals underwent an acclimatization period in chronic husbandry (day minus 14 until day 0). On day 0, the animals received either saline or paclitaxel for the first time, followed by intravital fluorescence microscopy (IVM). Administration of saline or paclitaxel was repeated 6 times until day 11, with a second IVM analysis on this last day. In the control group (group 1), all animals received intraperitoneal injections of saline 180 min prior to IVM. In all treatment groups, paclitaxel was injected at time point 0 min. Then IVM assessment was performed as follows: immediate 0 min (group 2), 60 min (group 3), 120 min (group 4), and 180 min after intraperitoneal administration (group 5). Additionally, we performed measurements in untreated animals (native group 6). Since results in this group were almost identical to those of the control group, we refrained from further listing.

All 31 animals (Figure 1) were weighed twice a week and examined every other day for general and neurological behavior during the 11 days of the experimental course. A persistent inability to consume sufficient fluids and food, accompanied by a sustained loss of >25% of initial weight, and apathy of the animals were defined as termination criteria [20].

### 2.2. Medication

Application of either paclitaxel (therapy) or saline (control) was performed in a standardized procedure as intraperitoneal injections in the lower abdomen. In the paclitaxel group, Taxomedac^®^ (Medac GmbH, Wedel, Germany) was used, and, for all saline injections, saline 0.9%, (B Braun AG, Melsungen; Germany) was used.

### 2.3. Microscopy

Anesthesia: For IVM the animals were anesthetized by intraperitoneal injection of 30 µL of 10% ketamine (100 mg/mL; Betapharm, Vechta, Germany) and 2% xylazine (Rompun 2%, 20 mg/mL, Bayer AG, Leverkusen, Germany) in a mixing ratio of 1:4. Thereafter, retroorbital intravenous injections of 20 µL of 5% FITC-dextran, MW 150 kDa, (Sigma Aldrich, St. Louis, MO, USA) were performed to enhance the contrast of the microvascular network in the ear. The dye remains within the intravascular space for 4 h and is eliminated from the circulation by both the liver and the kidneys. Furthermore 50 µL of 0.5% rhodamine 6G (Sigma Aldrich, St. Louis, MO, USA) was injected for staining leukocytes and 30 µL of bisbenzimide (Hoechst 33342, 10 µmol/kg) for staining apoptotic cells. Subsequently, the animals were placed in the lateral position on a 37 °C heated transparent stage to maintain a body temperature of 38 °C. The ear to be investigated was stretched with its ventral surface down on the stage [20].

Intravital fluorescence microscopy (IVM): High-resolution multi-fluorescence microscopy was performed using the Axiotech vario microscope (Carl Zeiss, Jena, Germany) equipped with a 100 W halogen lamp and filter sets for the colors blue (excitation/emission 465–495 nm/>505 nm), green (510–560 nm/>575 nm), and ultraviolet (340–380 nm/>400 nm) using epi-illumination Colibri 7n (Carl Zeiss, Jena, Germany). By the use of water-immersion objectives Achroplan ×20/0.50 W and ×63/0.95 W (Carl Zeiss, Jena, Germany), final magnifications of ×200 and ×630 were achieved. Images were recorded at a rate of 30 frames per second by means of the charge-coupled DVD recorder DMR-EX99V (Panasonic, Osaka, Japan). The images were transferred to a DVD system for subsequent off-line analysis using the computer-assisted image analysis system Cap-Image (Dr. Zeintl, Engineering Office, Dreieich, Germany) [25]. The duration of continuous light exposure per observation area was limited to 60 sec to avoid phototoxic effects [20].

Off-line microcirculatory analysis: Photoprints at a low magnification illustrating the areas of interest allowed the repetitive assessment of all the microcirculatory parameters in identical observation fields along time. Due to the uniqueness and consistency of the microcapillary branching pattern, there was never any problem with identification. The leukocyte–endothelial cell interaction was assessed in two postcapillary venules as described by Vollmar et al. [26]. Next to these vessels, four areas served for the analysis of FCD and the number of condensed nuclei. The functional capillary density was defined as the total length of capillaries continuously perfused with red blood cells per observation area and is given in [cm/cm^2^] [27]. Apoptosis, which is associated with condensation, fragmentation, and crescent formation of their nuclear chromatin, was analyzed by counting the number of cells stained with bisbenzimid [26,28]. The flow behavior of the leukocytes was analyzed with respect to free floating, rolling, and adherent leukocytes, which were easily identified by their rhodamine 6G staining in contrast to non-stained erythrocytes [29]. Rolling leukocytes were defined as those cells moving along the venular wall at a velocity of less than 40% of that of leukocytes at the centerline and expressed as percentage [%] of the total leukocyte flux [n/min]. Venular leukocyte adherence was defined as the number of leukocytes not moving or detaching from the endothelial lining of the vessel wall during an observation period of 20 sec and expressed as non-moving cells per endothelial surface [n/mm^2^] [29]. Thrombocytes were also stained by rhodamine 6G and could be differentiated by their much smaller size compared with leukocytes. The arteriolar and venular diameters were measured via the Cap-Image computer-assisted image analysis system introduced by Klyscz et al. [25]. The microvascular permeability was assessed by the venous leakage of FITC-dextran and analyzed densitometrically by the ratio of extra- to intravascular fluorescence intensity [20].

### 2.4. Statistical Analysis

Statistical analyses were performed using Graph Pad Prism 8.4.3 (Graph Pad Software, San Diego, CA, USA). The number of animals per group was determined by a power calculation [30]. In the case of a normal distribution, the data are expressed as mean ± SD (standard deviation). In the absence of a normal distribution, the data are expressed as median with 25th and 75th percentiles and whiskers indicating minimum and maximum values. The data were analyzed by One-way ANOVA following the Holm–Sidak’s multiple comparisons test. The results were considered significant at *p* < 0.05.

### 2.5. Experimental Protocol

Before the initiation of the experimental protocol, all the animals underwent seven days of acclimatization to humans by daily handling, as described earlier [20].

Intravital microscopy was performed on all the animals at day 0 and 11. The animals were randomized in a blinded manner into a total of six groups: four treatment groups, in which all animals received paclitaxel; one control group treated with saline, in which the animals received a total of 6 cycles of therapy on days 0, 2, 4, 7, 9 and 11; and one native group as a validation group, which was not treated at all (Figure 1). Accordingly, after randomization, all the animals were given an identification that did not indicate any group affiliation. The control group received intraperitoneal saline at time point 0 min, and the IVM was assessed at time point 180 min (group 1). In all the treatment groups, paclitaxel was injected at time point 0 min. Then IVM assessment was graded between the groups as follows: immediate measurement after administration of paclitaxel (group 2), 60 min (group 3), 120 min (group 4), and 180 min following intraperitoneal administration (group 5). Additionally, we performed measurements in untreated animals (native group) to rule out that a single peritoneal stimulus with saline solution already leads to microcirculatory changes. The native group did not differ from the group with saline solution and was therefore not considered further and was not included in the final analysis.

## 3. Results

Thirty-one animals were included in this study. None of them showed a relevant loss of weight during the entire study (Table 1). Seven animals died during the second anesthesia for intravital microscopy from respiratory and circulatory arrest after the completion of treatment on day 11. The number of deaths was evenly distributed between the groups. Therefore, only 24 animals from groups 1–5 could be fully evaluated. Since the analysis revealed highly significant differences between groups in the main target variable “functional capillary density” at all the measurement time points, it was decided not to conduct any additional experiments to compensate for these losses in the sense of the commonly accepted 3Rs [31].

### Intravital Fluorescence Microscopic Analysis

**Figure 2:**
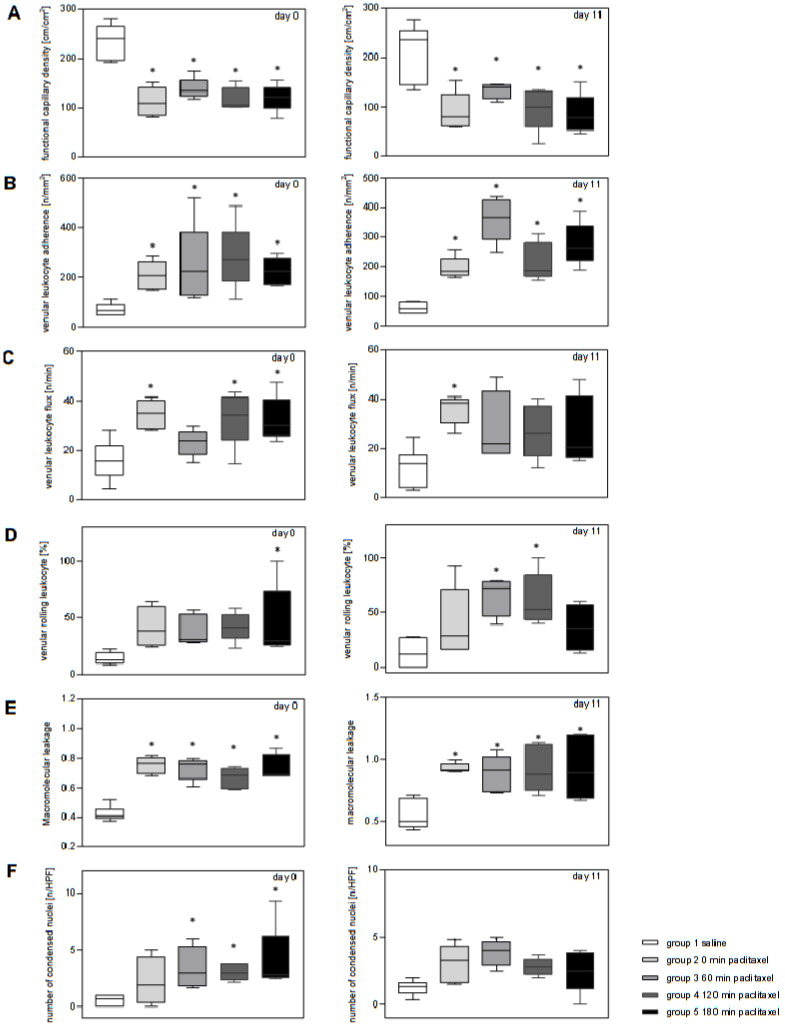
Quantitative analysis of intravital fluorescence microscopy with (**A**) functional capillary density in [cm/cm^2^], (**B**) venular leukocyte adherence [n/mm^2^], (**C**) venular leukocyte flux [n/min], (**D**) venular rolling leukocyte [%], as well as (**E**) macromolecular leakage and (**F**) bisbenzimide-stained condensed nuclei as [n/HPF], at the ear of paclitaxel- and saline-treated hairless SKH1-hr mice. IVM was performed on days 0 and 11: group 1 (saline), 180 min after the application of saline; group 2, directly after the application of chemotherapy; group 3, 60 min thereafter; group 4, 120 min thereafter; and group 5, 180 min thereafter. Data are expressed as median with 25th and 75th percentiles with whiskers indicating minimum and maximum values. * One-way ANOVA following Holm–Sidak’s multiple comparisons test revealed statistically significant differences between the paclitaxel and saline groups at *p* < 0.05.

The results of the intravital microscopy and microscopic cell analysis are listed in Table 1 and shown in Figure 2. On days 0 and 11 after the first administration of paclitaxel, a significant decrease in functional capillary density was found at each measurement time point compared with the saline (group 1) (Figure 2A).

As a sign of an inflammatory response to paclitaxel, the venous leukocyte adherence was significantly increased in all the paclitaxel treatment groups (group 2–5) on day 0 and 11 compared with that in group 1 (saline) (Figure 2B). In this context, the venous leukocyte flow was significantly increased in groups 2, 4, and 5 on days 0 and 11 in group 2 (Figure 2C). Compared with the saline (group 1), the venous rolling leukocytes, shown in Figure 2D, showed significant differences for group 5 on day 0 and for groups 3 and 4 on day 11.

As another sign of inflammation, intravital fluorescence microscopy showed a consistently increased macromolecular leakage exclusively in the paclitaxel groups, indicating an increased permeability of the venous endothelium (Figure 2E). This effect was significant at all time points after paclitaxel administration.

The quantification of cells showing condensation or fragmentation of nuclear chromatin revealed a higher number of apoptotic cells in the paclitaxel groups compared with that in the saline group (Figure 2F). This effect was significant on day 0 in groups 3, 4, and 5 (Figure 2F).

The results of other microcirculatory parameters such as arteriolar and venular diameters are presented in Table 1, showing no significant differences during the trial and between the treatment groups.

## 4. Discussion

We showed that both microcirculatory dysfunction and signs of inflammation start immediately, within seconds, after the administration of paclitaxel and that these pathological features last for at least 180 min.

Our results suggest that, in addition to the direct cyto- and neurotoxic effect of paclitaxel, inflammation triggered by a prolonged microcirculatory disturbance and reperfusion is another major contributor to the development of CIPN and musculoskeletal pain syndrome. It remains to be clarified whether the inflammatory reaction described here as being the result of reperfusion can be directly influenced by modifying the tissue temperature of the extremities or by other interventions. Furthermore, if so, the clinical consequences of prolonged cooling or compression therapy for CIPN and musculoskeletal pain syndrome need to be investigated.

The microcirculatory disturbances under chemotherapy demonstrated by various research groups are explained by different pathomechanisms [32,33,34]. Wang et al. defined oxaliplatin-induced CIPN as an ischemic phenomenon associated with reduced blood flow and an increased tendency toward thrombosis due to the formation of NETs [32]. An effect of the chemotherapeutic agent on the microscopic vascular nerve reticulum directly affecting the vasomotor tone has been described as another possible cause [33,34]. The results of our study support this theory.

However, with the IVM examination method we chose, we were only able to visualize the microcirculatory disturbance at the tissue level of the ear and not at individual nerves. However, the visualization of the temporal profile of the circulatory disturbance, with an immediate and persistent reduction in functional capillary density up to 3 h after drug administration, allows two major assumptions: on the one hand, the persistently reduced perfusion of the tissue may have caused direct cyto- and neurotoxic damage, and, on the other hand, this effect was aggravated by an additional reperfusion injury. Reperfusion generates a calcium overload in the cell with the formation of ROS and the subsequent activation of the MPT, which then contributes significantly to reperfusion injury [35]. The magnitude of tissue damage depends on several factors, such as the duration of ischemia, the extent of the ischemic body region, the type of tissue damaged, and the current functional activity of the tissue [36]. In accordance with this, we were also able to demonstrate significantly increased endothelial leakage as a sign of increased vascular permeability and direct endothelial toxicity in the paclitaxel group. We assume a direct cytotoxic effect, as this effect was observed immediately after the administration of the chemotherapeutic agent and with a persistence of at least 3 h. The adherence of leukocytes to the vascular endothelium further supports the idea of direct toxic effects and the induction of inflammation in endothelial cells [37]. In this context, we observed a significantly increased venous leukocyte adherence in the paclitaxel groups. This described cytotoxicity is also consistent with our intravital microscopic findings, which show clear signs of the activation of apoptosis early after the administration of the first paclitaxel cycle. With the native group (validation group), we were able to show that the mechanical intraperitoneal stimulus triggered by the saline treatment does not appear to produce any microcirculatory effect. This effect appears to be caused by the intraperitoneal application of paclitaxel.

From the multitude of neurotoxic chemotherapies, we chose the established taxan paclitaxel, which is widely used for many tumor entities and known for its high incidence of CIPN. We applied a cumulative dose of 30 mg/kg, which has already been identified as causing both microcirculatory dysfunction with inflammatory reaction and apoptosis [20], as well as neurological deficits [20,38]. We did not add a vehicle group with the pharmacological carrier cremophor, as no relevant microcirculatory dysfunction could be detected in this group in previous studies [20]. For intravital microscopy, we decided to use the SKh-1 mouse strain. We took advantage of the fin-like angioarchitecture of the pinna in this strain [24], which loses its fur after postnatal day 10 due to a genetic defect on chromosome 14, leaving the ear available as a peripheral measurement site.

Of course, our study also has clear limitations: We used the intraperitoneal route of administration, which is common in small animal models, whereas in humans the intravenous route of administration is standard clinical practice. The substance is absorbed through the peritoneal lining and into the bloodstream via the capillaries surrounding the peritoneum. This process is usually somewhat slower than intravenous administration. However, in this context, it is remarkable that, even by intraperitoneal application, the onset of microcirculatory dysfunction was immediate. Further, microcirculatory and metabolic effects were not specifically investigated here, which could give further information on the potential timing of preventive strategies. Another limitation is that we did not investigate microcirculation beyond 3 h. We chose this timeframe since, from our experience, the clinical appearance of peripheral microcirculatory dysfunction (pale, livid finger and toe tips) in patients lasts much shorter. This surprising experimental finding of such a prolonged effect needs further exploration, i.e., for how long these effects can be observed. Further, as neuropathological changes at a macroscopic level were not directly assessed in the present work, the final mechanistic connection between vascular and circulatory impairments and neurological changes remains to be demonstrated.

This investigation was associated with a surprisingly high mortality rate, which we interpret as a sign of the high invasiveness of this approach. We intensely discussed, whether additional experiments were justified to compensate for these losses, weighing sacrificing additional animal life against further gain of knowledge. Since analysis revealed highly significant differences between groups in the main target variable “functional capillary density” at all the measurement time points, we decided against additional experiments.

## 5. Perspectives

We therefore conclude that paclitaxel-induced microcirculatory disturbances and signs of local inflammation set in immediately after application and last at least for three hours. This suggests that options for prevention, or at least amelioration, could be potentially most successful if initiated in parallel to the induction of chemotherapy and continued for a longer timeframe (at least 3 h) after the end of application. Whether and to what extent the prolongation of preventive strategies can influence CIPN in the long run clinically needs to be studied in future investigations.

## Figures and Tables

**Table 1:** Microcirculatory parameters and body weight in hairless SKH1-hr mice, which were treated with either saline or paclitaxel for evaluation of CIPN. Values are given as mean ± SD. Two-way ANOVA and Tukey’s multiple comparisons test revealed no statistically significant differences.

Parameter	Group	Day 0	Day 11
arteriolar diameter [µm]	(1) 180 min saline	28.5 ± 5.0	36.3 ± 5.7
	(2) 0 min paclitaxel	27.9 ± 4.2	32.7 ± 3.4
	(3) 60 min paclitaxel	27.6 ± 4.7	31.6 ± 5.4
	(4) 120 min paclitaxel	30.1 ± 3.9	33.5 ± 6.2
	(5) 180 min paclitaxel	29.3 ± 4.9	31.3 ± 6.2
venular diameter [µm]	(1) 180 min saline	52.6 ± 9.0	58.1 ± 7.4
	(2) 0 min paclitaxel	63.6 ± 9.4	62.9 ± 6.2
	(3) 60 min paclitaxel	55.9 ± 9.8	49.7 ± 5.8
	(4) 120 min paclitaxel	62.6 ± 7.6	64.5 ± 8.4
	(5) 180 min paclitaxel	59.3 ± 10.6	58.0 ± 17.3
body weight [g]	(1) 180 min saline	21.1 ± 0.5	22.0 ± 0.2
	(2) 0 min paclitaxel	24.7 ± 3.7	24.4 ± 3.6
	(3) 60 min paclitaxel	24.7 ± 2.4	25.7 ± 2.7
	(4) 120 min paclitaxel	25.5 ± 1.5	26.3 ± 1.6
	(5) 180 min paclitaxel	24.4 ± 1.3	25.0 ± 1.1

## Data Availability

The original contributions presented in this study are included in the article. Further inquiries can be directed to the corresponding author.

## Abbreviations

ARRIVE	Animal Research Reporting of In Vivo Experiments
CIPN	Chemotherapy-induced peripheral neuropathy
FITC-dextran	Fluorescein isothiocyanate-labeled dextran
FCD	Functional capillary density
IVM	Intravital fluorescence microscopy
MPT	Mitochondrial permeability transition pore
MV	Molecular volume
NETs	Neutrophil extracellular traps
ROS	Reactive oxygen species

## References

[1] Lustberg M.B., Kuderer N.M., Desai A., Bergerot C., Lyman G.H. (2023). Mitigating long-term and delayed adverse events associated with cancer treatment: Implications for survivorship. Nat. Rev. Clin. Oncol..

[2] Salat K. (2020). Chemotherapy-induced peripheral neuropathy: Part 1-current state of knowledge and perspectives for pharmacotherapy. Pharmacol. Rep..

[3] Stubblefield M.D., Burstein H.J., Burton A.W., Custodio C.M., Deng G.E., Ho M., Junck L., Morris G.S., Paice J.A., Tummala S. (2009). NCCN task force report: Management of neuropathy in cancer. J. Natl. Compr. Cancer Netw..

[4] Banach M., Juranek J.K., Zygulska A.L. (2016). Chemotherapy-induced neuropathies—A growing problem for patients and health care providers. Brain Behav..

[5] Cioroiu C., Weimer L.H. (2017). Update on chemotherapy-induced peripheral neuropathy. Curr. Neurol. Neurosci. Rep..

[6] Flatters S.J.L., Dougherty P.M., Colvin L.A. (2017). Clinical and preclinical perspectives on Chemotherapy-Induced Peripheral Neuropathy (CIPN): A narrative review. Br. J. Anaesth..

[7] Seretny M., Currie G.L., Sana E.S., Ramnarine S., Grant R., MacLeod M.R., Colvin L.A., Fallon M. (2014). Incidence, prevalence, and predictors of chemotherapy-induced peripheral neuropathy: A systematic review and meta-analysis. Pain.

[8] Colvin L.A. (2019). Chemotherapy-induced peripheral neuropathy (CIPN): Where are we now?. Pain.

[9] Fallon M.T. (2018). Neuropathic pain in cancer. Br. J. Anaesth..

[10] Argyriou A.A., Bruna J., Marmiroli P., Cavaletti G. (2012). Chemotherapy-induced peripheral neurotoxicity (CIPN): An update. Crit. Rev. Oncol. Hematol.

[11] Bernhardson B.M., Tishelman C., Rutqvist L.E. (2007). Chemosensory changes experienced by patients undergoing cancer chemotherapy: A qualitative interview study. J. Pain Symptom Manag..

[12] Staff N.P., Grisold A., Grisold W., Windebank A.J. (2017). Chemotherapy-Induced Peripheral Neuropathy: A Current Review. Ann. Neurol..

[13] D’Souza R.S., Alvarez G.A.M., Dombovy-Johnson M., Eller J., Abd-Elsayed A. (2023). Evidence-Based Treatment of Pain in Chemotherapy-Induced Peripheral Neuropathy. Curr. Pain Headache Rep..

[14] Reeves B.N., Dakhil S.R., Sloan J.A., Wolf S.L., Burger K.N., Kamal A., Le-Lindqwister N.A., Soori G.S., Jaslowski A.J., Kelaghan J. (2012). Further data supporting that paclitaxel-associated acute pain syndrome is associated with development of peripheral neuropathy: North Central Cancer Treatment Group trial N08C1. Cancer.

[15] Loprinzi C.L., Reeves B.N., Dakhil S.R., Sloan J.A., Wolf S.L., Burger K.N., Kamal A., Le-Lindqwister N.A., Soori G.S., Jaslowski A.J. (2011). Natural history of paclitaxel-associated acute pain syndrome: Prospective cohort study NCCTG N08C1. J. Clin. Oncol. Off. J. Am. Soc. Clin. Oncol..

[16] Meregalli C., Fumagalli G., Alberti P., Canta A., Carozzi V.A., Chiorazzi A., Monza L., Pozzi E., Sandelius Å., Blennow K. (2018). Neurofilament light chain as disease biomarker in a rodent model of chemotherapy induced peripheral neuropathy. Exp. Neurol..

[17] Meregalli C., Fumagalli G., Alberti P., Canta A., Chiorazzi A., Monza L., Pozzi E., Carozzi V.A., Blennow K., Zetterberg H. (2020). Neurofilament light chain: A specific serum biomarker of axonal damage severity in rat models of Chemotherapy-Induced Peripheral Neurotoxicity. Arch. Toxicol..

[18] Huehnchen P., Schinke C., Bangemann N., Dordevic A.D., Kern J., Maierhof S.K., Hew L., Nolte L., Körtvelyessy P., Göpfert J.C. (2022). Neurofilament proteins as a potential biomarker in chemotherapy-induced polyneuropathy. JCI Insight.

[19] Areti A., Yerra V.G., Naidu V.G.M., Kumar A. (2014). Oxidative stress and nerve damage: Role in chemotherapy induced peripheral neuropathy. Redox Biol..

[20] Reuter S., Bajorat R., Müller-Graf F., Zitzmann A.R., Müller V., Pickhardt A.L., Reuter D.A., Böhm S.H., Vollmar B. (2025). The role of microcirculatory dysfunction during paclitaxel treatment as a critical co-factor for the development of chemotherapy-induced peripheral neuropathy. Geburtshilfe Und Frauenheilkd..

[21] TierSchG. https://www.gesetze-im-internet.de/tierschg/.

[22] European Animal Research Association EU Regulations on Animal Research. https://www.eara.eu/animal-research-law.

[23] Barker J.H., Hammersen F., Bondàr I., Uhl E., Galla T.J., Menger M.D., Messmer K. (1989). The hairless mouse ear for in vivo studies of skin microcirculation. Plast. Reconstr. Surg..

[24] Eriksson E., Boykin J.V., Pittman R.N. (1980). Method for in vivo microscopy of the cutaneous microcirculation of the hairless mouse ear. Microvasc. Res..

[25] Klyscz T., Jünger M., Jung F., Zeintl H. (1997). Cap image—A new kind of computer-assisted video image analysis system for dynamic capillary microscopy. Biomed. Tech..

[26] Vollmar B., El-Gibaly A.M., Scheuer C., Strik M.W., Bruch H.P., Menger M.D. (2002). Acceleration of cutaneous wound healing by transient p53 inhibition. Lab. Investig..

[27] Arora N., Islam S., Wafa K., Zhou J., Toguri J.T., Cerny V., Lehmann C. (2017). Evaluation of iris functional capillary density in experimental local and systemic inflammation. J. Microsc..

[28] Vollmar B., Morgenthaler M., Amon M., Menger M.D. (2000). Skin microvascular adaptations during maturation and aging of hairless mice. Am. J. Physiol. Heart Circ. Physiol..

[29] Sehnert B., Gierer P., Ibrahim S., Kühl A., Voll R., Nandakumar K.S., Holmdahl R., Hallmann R., Vollmar B., Burkhardt H. (2006). Modulation of granulocyte-endothelium interactions by antileukoproteinase: Inhibition of anti-type II collagen antibody-induced leukocyte attachment to the synovial endothelium. Arthritis Res. Ther..

[30] Lenth R.V. (2007). Statistical power calculations. J. Anim. Sci..

[31] NSW Department of Primary Industries and Animal Research Review Panel Replacement, Reduction and Refinement (3 Rs). https://www.dpi.nsw.gov.au/dpi/animals/animal-ethics-infolink/three-rs.

[32] Wang C.Y., Lin T.T., Hu L., Xu C.J., Hu F., Wan L., Yang X., Wu X.F., Zhang X.T., Li Y. (2023). Neutrophil extracellular traps as a unique target in the treatment of chemotherapy-induced peripheral neuropathy. EBioMedicine.

[33] Peterson E.R., Crain S.M. (1982). Nerve growth factor attenuates neurotoxic effects of Taxol on spinal cord-ganglion explants from fetal mice. Science.

[34] Wiernik P.H., Schwartz E.L., Strauman J.J., Dutcher J.P., Lipton R.B., Paietta E. (1987). Phase I clinical and pharmacokinetic study of Taxol. Cancer Res..

[35] Kalogeris T., Baines C.P., Krenz M., Korthuis R.J. (2016). Ischemia/Reperfusion. Compr. Physiol..

[36] Burda R., Burda J., Morochovič R. (2023). Ischemic Tolerance-A Way to Reduce the Extent of Ischemia-Reperfusion Damage. Cells.

[37] Panés J., Perry M., Granger D.N. (1999). Leukocyte-endothelial cell adhesion: Avenues for therapeutic intervention. Br. J. Pharmacol..

[38] Toma W., Kyte S.L., Bagdas D., Alkhlaif Y., Alsharari S.D., Lichtman A.H., Chen Z.J., Del Fabbro E., Bigbee J.W., Gewirtz D.A. (2017). Effects of paclitaxel on the development of neuropathy and affective behaviors in the mouse. Neuropharmacology.

